# Iron accumulation in the corpus callosum: a quantitative MRI study in relapsing-remitting multiple sclerosis

**DOI:** 10.1007/s11845-026-04309-y

**Published:** 2026-04-09

**Authors:** Ali Al-Radaideh, Mustafa M. Almuqbel, Sarah Abousrafa, Abdullah Radaideh, Majed Hbahbih

**Affiliations:** 1 Department of Medical Radiography, College of Health Sciences, University of Doha for Science and Technology, Doha, Qatar; 2 Department of Medical Imaging, Faculty of Applied Medical Sciences, The Hashemite University, Zarqa, Jordan; 3 School of Medicine, University of Jordan, Amman, Jordan; 4 Neurology Section, Internal Medicine Department, Jordan University Hospital, Amman, Jordan; 5 Department of Internal Medicine, Neurology Section, King Hussein Medical Center, Jordanian Royal Medical Services, Amman, Jordan; 6 Department of Radiology, King Hussein Medical Center, Jordanian Royal Medical Services, Amman, Jordan; 7 New Zealand Brain Research Institute, Christchurch, New Zealand

**Keywords:** Iron accumulation, Corpus callosum, Multiple sclerosis, Magnetic resonance imaging, Quantitative analysis

## Abstract

**Purpose:**

This study quantitatively evaluated iron accumulation in the normal-appearing corpus callosum (CC) of multiple sclerosis (MS) patients using multi-parametric magnetic resonance imaging (MRI), including quantitative susceptibility mapping, MTR, T1/T2 mapping, and volumetry, and examined associations with white matter lesions, disease duration (DD), and Expanded Disability Status Scale (EDSS).

**Methods:**

Brain MRI of 58 patients with RRMS (32 females, 26 males; mean age 37 ± 9.07 years; age range 19-58 years) along with 58 age and gender-matched healthy controls (HCs) (27 females, 31 males; mean age 38 ± 8.8 years; age range 19-57 years) were extracted and analyzed. 3D-high resolution T1, T2-FLAIR, and T2* images, along with MTR, T1 and T2 maps were used for volumetric segmentation, WM lesions extraction, iron calculation, MTR, T1 and T2 relaxation times of CC, respectively.

**Results:**

RRMS patients exhibited significantly higher iron concentrations across all corpus callosum (CC) regions compared with healthy controls (*p* < 0.01). Subregional analyses revealed that associations between iron concentration and imaging variables were confined primarily to anterior CC segments, most notably the mid-anterior CC, with no associations observed for disease duration, EDSS, or white matter lesion load. Among MRI metrics, iron achieved the highest discriminative performance (AUC = 0.74; sensitivity 100%, specificity 50%), while volume provided more balanced classification (AUC = 0.66).

**Conclusions:**

Quantitative MRI revealed iron accumulation in the corpus callosum of RRMS patients compared to HCs. Iron was the most sensitive marker for distinguishing patients, supporting its potential as an MS biomarker.

## Introduction

The corpus callosum (CC) is the largest white matter (WM) bundle of commissural connections within the human brain [1]. Morphologically, distinct components such as the rostrum, genu, body, and splenium characterize the CC [2]. The CC is an excellent structure in evaluating injury to the normal appearing white matter (NAWM), due to its high fiber orientation coherence and reasonably well-defined rostral and caudal borders [3].

Multiple sclerosis (MS) is a long-lasting autoimmune disorder marked by the breakdown of myelin, progressive neurodegeneration, and subsequent neurological deficits, including gliosis and loss of neurons. T cells are key contributors to the pathological process, as they initiate and sustain demyelination [4]. Disruption of iron balance is increasingly recognized as a major factor in the pathophysiology of MS [5]. When iron levels become excessive, they contribute to neuronal injury through pathways such as ferroptosis and alterations in immune activity [6]. High iron concentrations stimulate inflammatory responses by driving macrophages toward a pro-inflammatory state and shifting microglia into a similarly activated phenotype. This inflammatory shift heightens oxidative stress, undermines mitochondrial integrity, and increases the generation of reactive oxygen species, all of which contribute to neuronal damage [7]. Pro-inflammatory cytokines, particularly IL-6, further shape iron regulation and facilitate the expansion of Th17 cells, thereby worsening inflammation within the CNS [8, 9]. During MS, both macrophages and microglia accumulate iron, which reinforces neuroinflammation and promotes demyelination. On the other hand, insufficient iron can impair normal immune processes and modify T-cell behavior [10]. Together, these effects emphasize the importance of maintaining iron homeostasis in the onset and progression of MS.

MS disease causes inflammatory demyelination and axon damage in the CC. Notably, the CC is considered as a common brain element affected by the MS lesions, often detected through diagnostic methods [11]. Normally, CC is relatively resistant to age-related changes in healthy individuals, which makes its morphology a promising biomarker for MS pathology [12, 13].

Atrophy of the CC and the presence of demyelinating lesions within callosal and subcallosal regions are well-documented features of multiple sclerosis [11, 14, 15]. Beyond these established structural changes, our study provides new insight by demonstrating measurable iron accumulation within the CC, a phenomenon that has not been widely characterized. This observation extends current understanding of CC involvement by linking callosal pathology not only to demyelination and atrophy but also to iron-related neuroinflammatory processes.

Given the emerging significance of iron dysregulation in MS, quantifying MRI-derived indices capable of capturing iron-related alterations in this structure is essential for advancing the understanding of CC pathology. Therefore, the primary aim of this study was to quantitatively characterize the normal-appearing CC (apparently lesions free) using quantitative susceptibility mapping (QSM) as the principal iron-sensitive measure, alongside volumetry, magnetization transfer ratio (MTR), T1 mapping, and T2 mapping. A secondary aim was to explore how iron content within the CC, as measured by QSM, relates to other MRI-derived microstructural indices, providing further insight into the interplay between iron accumulation, myelin integrity, and tissue composition. Additionally, the association between CC quantitative MRI metrics and clinical status, white matter lesion burden, and physical disability as measured by the Expanded Disability Status Scale (EDSS) was also evaluated.

## Materials and methods

### Study population

Brain MRI scans of 58 patients with relapsing-remitting multiple sclerosis (RRMS) (32 females, 26 males; mean age 37 ± 9.07 years; age range 19–58 years) along with 58 age and gender-matched healthy controls (HCs) (27 females, 31 males; mean age 38 ± 8.8 years; age range 19–57 years) were analyzed. The characteristics of the two study groups are summarized in Table 1. The selection criteria of the MRI scans were as follows: brains MRI images of participants who were aged between 18 and 65 years at the time of scanning, diagnosed with RRMS according to the revised 2010 McDonald’s criteria [16], and have no prior history of other central nervous system diseases, including demyelinating and neurodegenerative diseases, brain tumor or surgery, head injury, or cerebrovascular disease. Brain MRI scans showing MS lesions within the CC were also excluded from the analysis, as such lesions can significantly impact the measured metrics, thereby introducing potential confounding effects on the overall results. The current study obtained approval from the institutional ethics committee (No. 3/7/2021/2022) and was conducted in compliance with The Code of Ethics of the World Medical Association (Declaration of Helsinki). EDSS scores, MS disease duration (DD), and number of relapses were also extracted for all RRMS patients. RRMS patients received one of two established disease-modifying therapy (DMT) strategies. Patients with mild to moderate disease activity were treated with interferon beta-1a (Rebif) 44 µg three times per week or interferon beta-1b (Betaferon) 250 µg every other day, both administered subcutaneously. Patients with higher disease activity or an inadequate response to first-line therapies received natalizumab 300 mg intravenously every four weeks. This treatment stratification reflects standard clinical practice based on disease severity and therapeutic response profiles. Prior to the MRI scanning, these neurological assessments were performed previously by a qualified consultant neurologist using the standardized scoring system in Neurostatus [17].

**Table 1:** Characteristics of relapsing-remitting multiple sclerosis (RRMS) and healthy controls (HCs)

	Subjects	
	HCs	RRMS
Number	58	58
Sex (female/male)	(27/31)	(32/26)
Age range (years)	19-57	19-58
Mean age ± SD (years)	38 ± 8.8	37 ± 9.7
EDSS range	-	1-8.5
Mean EDSS ± SD	-	4.78 ± 1.98
DD range (years)	-	1-17.8
Mean DD ± SD (years)	-	6.8 ± 4.1
Number of relapses (range)	-	1-8
Mean number of relapses ± SD	-	3.6 ± 1.83

*RRMS,* relapsing-remitting multiple sclerosis; *HCs,* healthy controls; *SD,* standard deviation; *EDSS,* Expanded Disability Status Scale; and *DD,* Disease Duration

### Magnetic resonance imaging (MRI)

Extracted brain MRI scans were previously collected using 3 Tesla (T) Trio system (Siemens Medical Solutions). The imaging protocol comprised the following imaging sequences: a 3D T2-weighted Fluid Attenuated Inversion Recovery (FLAIR) with 1 mm^3^ isotropic spatial resolution; reconstruction matrix = 256 × 256; TE = 309 ms; TR = 5000 ms; inversion time = 1650 ms; flip angle = 90^o^; scan time = 5:52 min; a 3D T1-weighted Magnetization Prepared–Rapid Acquisition Gradient Echo (MPRAGE) with 1 mm^3^ isotropic spatial resolution; reconstruction matrix = 224 × 256; TE = 3.4 ms; TR = 1900 ms; inversion time = 900 ms; flip angle = 9^o^; scan time = 5:53 min; a 3D T2*-weighted gradient echo sequence with 1 mm^3^ isotropic spatial resolution, reconstruction matrix = 256 × 256; TE = 20 ms; TR = 33 ms; flip angle = 15^o^; scan time = 5:30 min; a 3D turbo FLASH sequence (with and without MT RF pulse) with 1 × 1 × 2 mm^3^ spatial resolution; reconstruction matrix = 256 × 256; TE = 5 ms; TR = 60 ms; flip angle = 10^o^; scan time = 3.35 min; the T1 maps were created from 3D-T1 weighted images acquired at 8 different inversion times(130, 400, 250, 600, 900, 1200, 1600, 2200 ms) with 1 × 1 × 3 mm^3^ spatial resolution; reconstruction matrix = 256 × 256; TE = 2.28 ms; TR = 8000 ms; flip angle = 9^o^; scan time per inversion time = 2.26 min; and T2 maps were generated from multi-echo turbo spin-echo images acquired at echo times (20, 40, 60, 80, 100 ms) with Echo Train Length (ETL) = 25; spatial resolution = 1.4 × 1.4 × 5 mm³; reconstruction matrix = 160 × 160; TR = 3000 ms; flip angle = 90°; ; scan time = 2:55 min.

### Image post-processing and analysis

All image post-processing and analysis steps were described in detail in previous publications [17, 18]. However, the following methodology sections will summarize those steps.

#### Volumetric segmentation of CC parts

**Fig. 1:**
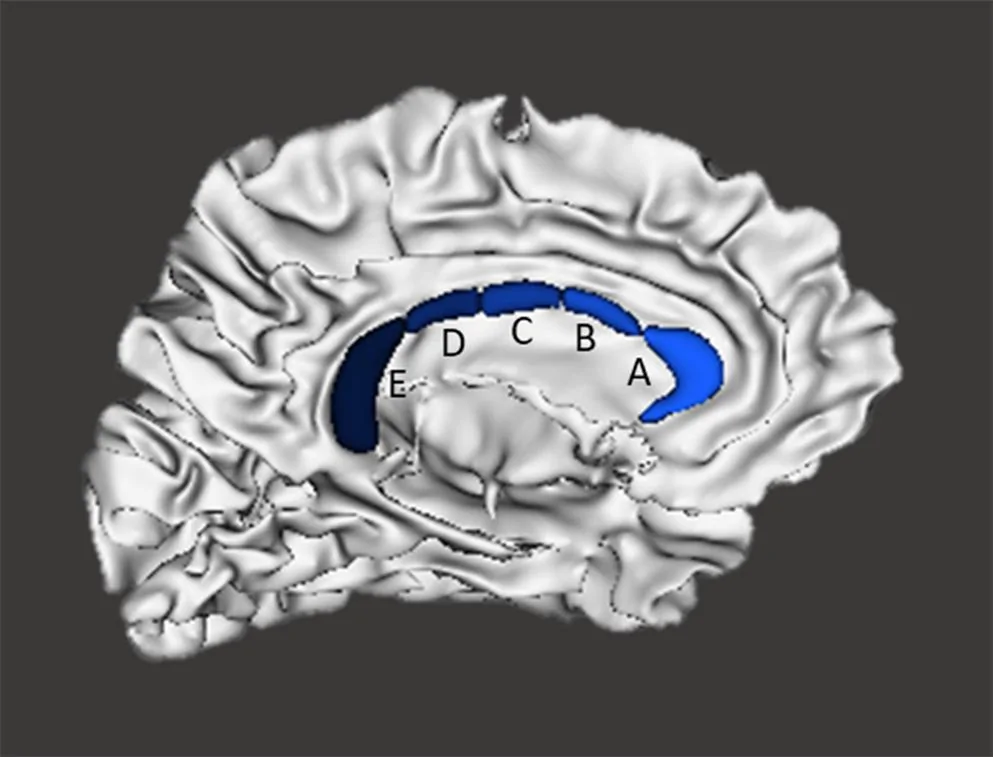
Corpus callosum segmentation in FreeSurfer. A) Anterior, B) Mid-Anterior, C) Central, D) Mid-Posterior, and E) Posterior

The volumetric segmentations of CC parts were performed with the FreeSurfer image analysis suite [19–21]. The CC was segmented into five parts (CC_Posterior, CC_Mid_Posterior, CC_Central, CC_Mid_Anterior, and CC_Anterior) followed by the calculation of whole CC volume, which was then normalized to the estimated total intracranial volume (eTICV) to account for differences in individual brain sizes (Fig. 1). The segmented CC parts were mapped (converted) from the conformed FreeSurfer space to the native anatomical space “rawavg” space, thresholded (using the CC parts’ label values provided in the FreeSurfer’s Look-Up-Table, LUT) and binarized to extract the corresponding anatomy from the subsequent iron, MTR, T1, and T2 maps.

#### Calculation of iron concentration

The T2*-weighted images were spatially co-registered to the T1-weighted MPRAGE using the FLIRT linear registration algorithm in FSL. The co-registered phase images were unwrapped in Prelude (in FSL) and high pass filtered to remove the effects of large-scale background fields [22]. For each CC part, the phase differences relative to that of the cerebrospinal fluid (CSF) in the lateral ventricles were converted to iron concentration according to the equation proposed by Haacke; 0.276 radians of the phase difference to be equivalent to 240 µg Fe/g tissue [23]. The phase values of CSF were used as a reference for iron calculation because it is assumed that CSF contains a negligible amount of MR-visible iron. Iron calculations were performed as described in previous work [17].

**Fig. 2:**
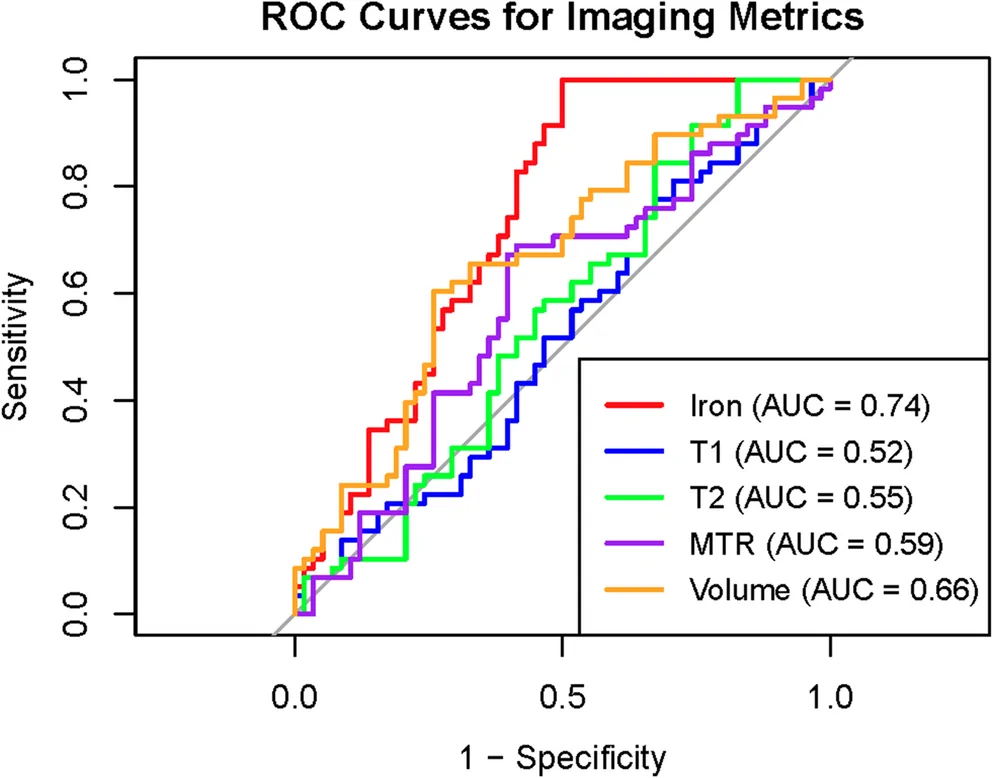
ROC curves for MRI-derived metrics in distinguishing RRMS patients from healthy controls. Iron and volume showed the highest discriminative performance, while T1, T2, and MTR had limited classification value

#### Calculation of the MTR values

The baseline volume of the MT sequence (MT RF pulse ‘off’) and the MT volume (MT RF pulse ‘ON’) were spatially co-registered to the T1-weighted MPRAGE using the FLIRT linear registration algorithm in FSL. The cost function “mutual information” with a low number (100) of bins was used due to the difference in contrast between the MPRAGE image and reference scan. The MTR maps were then calculated on a voxel-by-voxel basis using to the following formula:

$$\:MTR=\:\frac{\left(Volume\:\right(no\:MT)-\:Volume\:(with\:MT)}{Volume\:\left(no\:MT\right)}$$

#### Calculation of T1 relaxation times

Rigid-body image registration using the FLIRT linear registration algorithm in FSL was used to spatial co-register the eight volumes of sMPRAGE images to the high-resolution, T1-weighted MPRAGE. The co-registered images were then imported into JIM software (Jim version 7; Xinapse Systems) in which the T1 maps were fitted on a voxel-by-voxel basis.

**Fig. 3:**
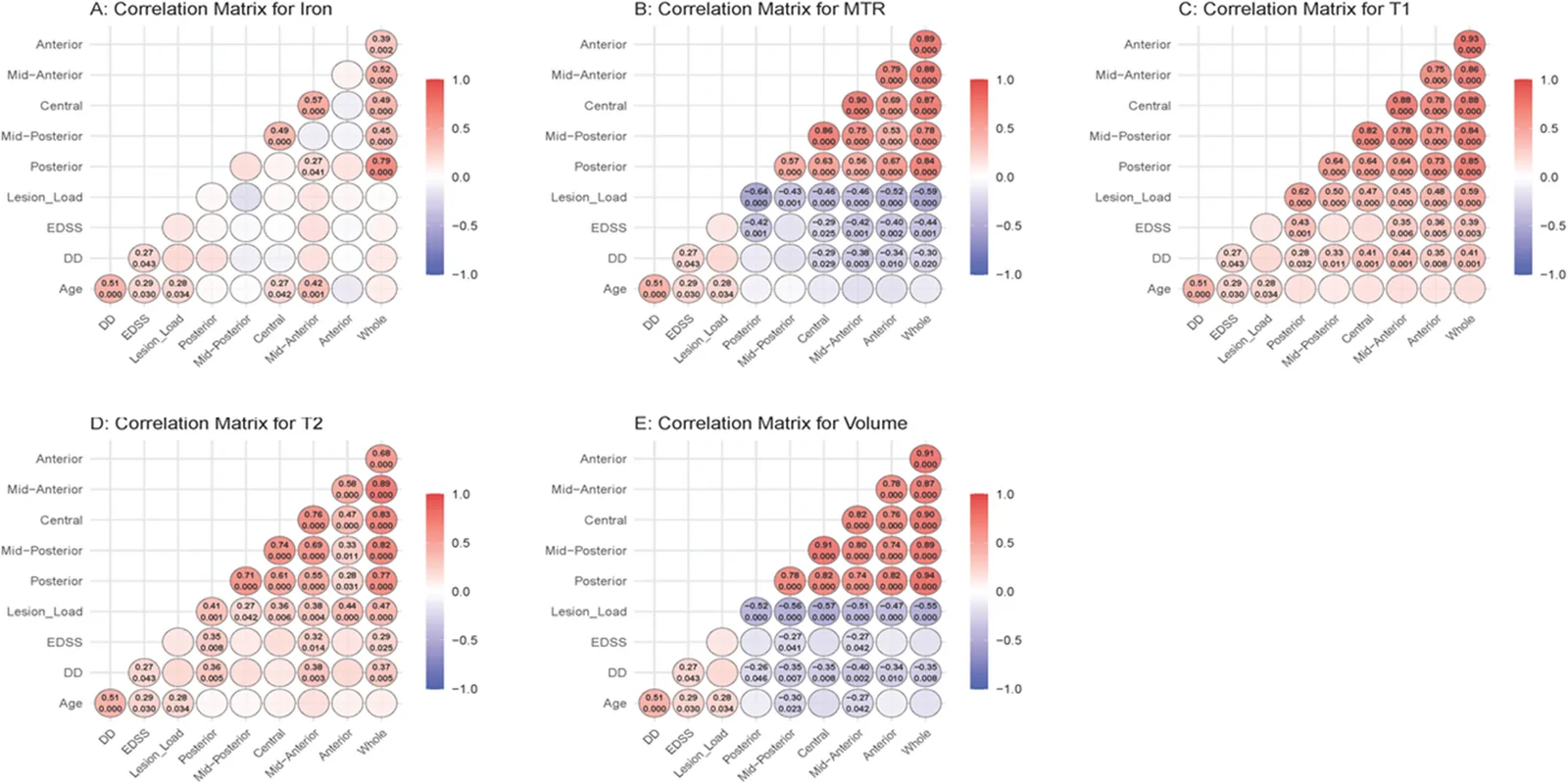
Pearson correlation coefficient matrices for the five MRI-derived metrics: Iron (panel **A**), MTR (panel **B**); T1-map (panel **C**); T2-map (panel **D**); and volume (panel **E**). The color gradient indicates the correlation strength (blue for negative and red for positive). To simplify the graphical representation of the correlation matrices, only the statistically significant (*p* < 0.05) relations are displayed

#### Calculation of T2 relaxation times

T2 maps were automatically calculated from the multi-echo (5 echoes), T2 weighted Turbo Spin echo sequence.

#### Calculation of WM-T2 lesion load

White matter T2-hyperintense lesion volume measurements were obtained using a semi-automated segmentation technique based on the Fuzzy Connectedness algorithm [24] in Jim software (Jim version 7; Xinapse Systems, Northants, England) as described in previous work [17]. In summary, the T2-weighted FLAIR images were spatially co-registered to the T1-weighted MPRAGE images in Jim. The registered T2-FLAIR images were used to manually define “seed” points in the center of each MS lesion and save them for each patient. “MS Finder” tool in Jim was then used to delineate the MS lesions, based on the Fuzzy Connectedness algorithm [24] using the T1 and T2-weighted images, “seed” points, and the “lesion prior probability”.

### Statistical analyses

All statistical analyses were conducted using R Statistical Software (v4.4.0; R Core Team 2024). Normality of the data was tested prior to analysis. Group differences in the quantitative metrics of various CC parts between MS and HCs were evaluated. Receiver Operating Characteristic (ROC) analysis was performed for each MRI-derived metric to evaluate its ability to discriminate between MS patients and healthy controls. Prior to analysis, all metric values were standardized (z-scores) to place them on a common scale with mean = 0 and standard deviation = 1, ensuring fair comparison across measures with different units and ranges. ROC curves were generated, and the Area Under the Curve (AUC) with 95% confidence intervals was calculated to quantify overall discriminative ability. The optimal cutoff for each metric was determined using Youden’s index, which maximizes the sum of sensitivity and specificity. Sensitivity and specificity values at these cutoffs were also reported to aid interpretation of diagnostic performance. Multivariate Linear Regression was performed to examine the independent associations between iron concentration and imaging variables (MTR, T1 relaxation time, T2 relaxation time, normalized volume, white matter lesion load), with and without age adjustment, across the whole CC and its subregions. The relationships among other variables (DD, EDSS, and white matter lesion load) stratified by CC MRI-derived metrics (iron, MTR, T1, T2, and volume) were evaluated using univariate correlation analysis. Pearson’s correlation coefficients and corresponding p-values were computed using the “rcorr” function from the “Hmisc” package [25]. Correlation heatmaps were generated for each metric using the “ggplot2” package [26], with a color gradient indicating correlation strength (blue for negative, red for positive), correlations were considered statistically significant if *p* < 0.05. To focus on unique pairwise relationships, the lower triangle and diagonal of the correlation matrix were masked, and only significant correlations were presented.

## Results

While the RRMS patients recorded significantly (*p* < 0.01) higher iron concentration than HCs in entire CC, only the mid-posterior, central, and mid-anterior portions of the CC of RRMS patients showed significantly (*p* < 0.05) reduced MTR values than those of the HCs. In addition, the anterior part of the CC in patients showed remarkably (*p* < 0.05) higher T2 relaxation times relative to HCs. In contrast, no significant differences in T1 relaxation times were identified between the CC parts of RRMS patients and HCs. A significant statistical difference (*p* < 0.01) was observed between RRMS patients and HCs across all CC regions, with RRMS patients showing significantly smaller CC volumes than HCs (Table 2).

Among the five MRI-derived metrics assessed for discriminating MS patients from healthy controls, iron showed the highest discriminative ability with an AUC of 0.74 (95% CI: 0.65–0.83), indicating acceptable discrimination. At its optimal standardized cutoff (− 0.83), it achieved perfect sensitivity (100%) but moderate specificity (50%), meaning it successfully identifies all MS cases but misclassifies half of healthy controls. Volume also demonstrated some discriminative ability (AUC = 0.66, 95% CI: 0.56–0.76) with a more balanced sensitivity (60%) and higher specificity (74%), suggesting it may be better suited for excluding healthy controls at its cutoff. In contrast, the other metrics T1, T2, and MTR, had AUC values close to 0.5–0.58 with confidence intervals crossing 0.5, indicating poor to no discriminative value in this sample. Their low specificities at the chosen thresholds further limit their utility for classification in this context (Fig. 2).

Multivariate linear regression was performed to examine the independent associations between iron concentration and imaging variables (MTR, T1 relaxation time, T2 relaxation time, normalized volume, white matter lesion load), with and without age adjustment, across the whole CC and its subregions.

At the whole-CC level, the regression model explained minimal variance in iron concentration and was not statistically significant (R² = 0.033; adjusted R² = −0.060; *p* = 0.874). None of the imaging variables showed significant independent associations with iron concentration (all *p* ≥ 0.33). Inclusion of age resulted in only a marginal, nonsignificant increase in explained variance (R² = 0.049; adjusted R² = −0.063; *p* = 0.853), and age was not independently associated with iron concentration (β = 0.131, *p* = 0.371).

In the anterior CC, the multivariate model explained a modest proportion of variance (R² = 0.134; adjusted R² = 0.051) but was not statistically significant overall (*p* = 0.173). T2 relaxation time was the only imaging variable independently associated with iron concentration (β = 0.424, *p* = 0.037), while MTR, T1, normalized volume, and white matter lesion load were nonsignificant. After age adjustment, the association between T2 and iron remained significant (β = 0.399, *p* = 0.049), whereas age and all other variables were not independently associated.

In contrast, the mid-anterior CC demonstrated statistically significant models both before (R² = 0.247; adjusted R² = 0.174; *p* = 0.010) and after age adjustment (R² = 0.374; adjusted R² = 0.300; *p* < 0.001). T1 and T2 relaxation times were independently associated with iron concentration in both models (T1: β = −0.576 to − 0.586, *p* ≤ 0.004; T2: β = 0.538 to 0.525, *p* ≤ 0.023). Age was also independently associated with iron concentration in the age-adjusted model (β = 0.376, *p* = 0.002). MTR, normalized volume, and white matter lesion load were not significant predictors.

In the central CC, the unadjusted model was nonsignificant (R² = 0.073; adjusted R² = −0.016; *p* = 0.540). After age adjustment, overall model fit increased modestly (R² = 0.147; adjusted R² = 0.046) but remained nonsignificant (*p* = 0.211). Age was the only variable independently associated with iron concentration (β = 0.286, *p* = 0.041), while all imaging variables were nonsignificant.

For the mid-posterior CC, neither the unadjusted (R² = 0.100; adjusted R² = 0.013; *p* = 0.346) nor age-adjusted model (R² = 0.101; adjusted R² = −0.005; *p* = 0.467) was statistically significant. No imaging variable or age showed an independent association with iron concentration.

In the posterior CC, the model explained a modest proportion of variance (R² = 0.149; adjusted R² = 0.067) but was not statistically significant (*p* = 0.124). T1, T2, and normalized volume showed borderline but nonsignificant associations with iron concentration (*p* = 0.061–0.089). After age adjustment, model fit remained nonsignificant (R² = 0.154; adjusted R² = 0.054; *p* = 0.184), and no predictor, including age, reached statistical significance.

Across all regions, collinearity diagnostics indicated acceptable multicollinearity (VIFs within conventional thresholds), supporting model stability.

The univariate correlation results are presented in Fig. 3, which provides a heatmap representation of the strength of correlations between the various MRI metrics and clinical variables (DD, EDSS, and white matter lesion load), along with their corresponding statistical significance.

The analysis of iron content in the whole CC and its subregions revealed no significant correlations with the DD, EDSS, or white matter lesion load (Fig. 3, panel A).

Significant weak negative associations were observed between the DD and the MTR in all regions of the CC, except for the posterior regions (Fig. 3, panel B). Similarly, except for the mid-posterior region, EDSS scores exhibited significant weak to moderate negative associations with the MTR. All regions of the CC showed significant moderate to high negative correlations between the white matter lesion load and the MTR.

The analysis of relationship between the T1 relaxation times in the CC regions and the DD revealed significant weak to moderate positive associations across all CC regions (Fig. 3, panel C). Additionally, a significant weak to moderate positive association was detected between the EDSS scores and the T1 relaxation times in the posterior, mid-anterior, anterior parts, as well as the whole CC. A significant moderate to high positive correlation was identified between the T1 relaxation times and the white matter lesion load in all regions of the CC.

The correlation analysis between the T2 relaxation times in the CC and the DD and EDSS demonstrated significant weak positive associations in the posterior and mid-anterior regions (Fig. 3, panel D). A significant weak to moderate positive correlation was observed between the white matter lesion load and the T2 relaxation times in all CC regions.

Significant weak to moderate negative associations were observed between the DD and CC volume in all regions of the CC (Fig. 3, panel E). However, EDSS scores exhibited significant weak negative associations with the volumes of mid-posterior and mid-anterior regions of the CC. The correlation analysis between the white matter lesion load and CC volume across multiple regions revealed significant moderate negative correlations.

**Table 2:** Morphometric measures, iron concentration, MTR, and T1 and T2 relaxation times of the whole corpus callosum and its subregions

	Controls	RRMS	
	Mean ± SD	Mean ± SD	*p*-value
Mean iron concentration ± SD (µg)			
CC_posterior	9.39 ± 0.006	16.42 ± 0.008	<0.01
CC_mid-posterior	11.12 ± 0.007	20.26 ± 0.01	<0.01
CC_central	12.56 ± 0.007	23.88 ± 0.01	<0.01
CC_mid-anterior	10.19 ± 0.007	20.09 ± 0.009	<0.01
CC_anterior	6.98 ± 0.004	11.88 ± 0.006	<0.01
CC_whole	9.33 ± 0.003	16.71 ± 0.007	<0.01
MTR ± SD			
CC_posterior	0.36 ± 0.036	0.36 ± 0.039	>0.05
CC_mid-posterior	0.32 ± 0.042	0.28 ± 0.036	<0.05
CC_central	0.32 ± 0.043	0.29 ± 0.037	<0.05
CC_mid-anterior	0.32 ± 0.045	0.30 ± 0.045	<0.05
CC_anterior	0.33 ± 0.038	0.33 ± 0.042	>0.05
CC_whole	0.34 ± 0.032	0.33 ± 0.033	>0.05
T1 relaxation time ± SD (ms)			
CC_posterior	1006 ± 215	1007 ± 221	>0.05
CC_mid-posterior	1143 ± 243	1169 ± 261	>0.05
CC_central	1084 ± 245	1116 ± 277	>0.05
CC_mid-anterior	1088 ± 272	1143 ± 315	>0.05
CC_anterior	961 ± 184	978 ± 204	>0.05
CC_whole	1052 ± 180	1076 ± 208	>0.05
T2 relaxation time ± SD (ms)			
CC_posterior	289 ± 118	307 ± 100	>0.05
CC_mid-posterior	456 ± 225	488 ± 207	>0.05
CC_central	426 ± 198	462 ± 190	>0.05
CC_mid-anterior	400 ± 201	432 ± 173	>0.05
CC_anterior	235 ± 96	274 ± 140	<0.05
CC_whole	328 ± 132	358 ± 107	>0.05
Normalized CC volumes			
Normalized CC_posterior	0.047 ± 0.018	0.036 ± 0.017	<0.01
Normalized CC_mid-posterior	0.021 ± 0.01	0.012 ± 0.009	<0.01
Normalized CC_central	0.018 ± 0.008	0.010 ± 0.008	<0.01
Normalized CC_mid-anterior	0.02 ± 0.009	0.011 ± 0.006	<0.01
Normalized CC_anterior	0.043 ± 0.016	0.031 ± 0.015	<0.01
Normalized CC_whole	0.171 ± 0.064	0.134 ± 0.058	<0.01

*RRMS,* relapsing-remitting multiple sclerosis; *MTR,* magnetization transfer ratio; *SD,* standard deviation; *CC,* corpus callosum; *µg,* micro gram; *ms,* milli-second

## Discussion

There was an obvious increase in the iron concentrations in all parts of CC of RRMS patients compared with those of HCs. This suggests that iron accumulation occurred in CC during the relapsing-remitting phase of the MS disease. Iron changes in the splenium of the CC are in line with those found in a study by Eytan et al. (2014) [27]. The increased iron concentration observed in RRMS may reflect an ongoing inflammatory process or tissue damage, potentially contributing to disease progression.

This study demonstrates marked regional heterogeneity in the relationship between CC iron concentration and other MRI-derived imaging variables. At the whole-CC level, iron concentration was not independently associated with relaxometry measures, MTR, normalized volume, white matter lesion load, or age, indicating that global CC averaging obscures region-specific associations.

Subregional analyses revealed that associations between iron concentration and imaging variables were confined primarily to anterior CC segments, most notably the mid-anterior CC. In this region, iron concentration showed consistent independent associations with both T1 and T2 relaxation times and with age, whereas MTR, normalized volume, and white matter lesion load were not associated. In the anterior CC, a more limited association with T2 relaxation time was observed, while no other predictors were significant.

In contrast, central, mid-posterior, and posterior CC segments showed little to no independent association between iron concentration and imaging variables, with age emerging as a predictor only in the central CC.

Across all regions, white matter lesion load did not independently associate with iron concentration, suggesting that iron levels within the CC are not simply a surrogate for global lesion burden. Similarly, the absence of consistent associations with MTR and normalized volume indicates that iron concentration is largely independent of these commonly used markers when examined within the present multivariate framework.

Overall, these findings indicate that iron concentration and other imaging-derived biomarkers relationships within the CC are region-specific rather than uniform, with the mid-anterior CC showing the clearest associations and posterior regions showing minimal or no detectable relationships. This emphasizes the importance of subregional analyses and cautions against reliance on whole-structure measures when investigating iron-related processes in white matter.

No significant connections were detected between the CC iron concentrations, DD, EDSS, and white matter lesions. These findings suggest that the iron accumulation in the CC and its subregions does not appear to be associated with DD, disability progression, or the extent of white matter lesions. This could be attributed to the fact that the white matter fibers of the CC are very dense and highly coherent (mitigates the MRI dephasing phenomenon, which in turn makes it harder to depict iron-induced changes in the tissue), and these changes seem to be subtle at the relapsing-remitting phase of the MS disease. It is also possible that the role of iron in the CC is more complex and may involve factors not captured by the measures used in this study.

The present findings highlight iron content in the CC as the most informative MRI-derived metric for distinguishing MS patients from healthy controls. Among all measures evaluated, CC iron demonstrated the highest discriminative performance, with an AUC of 0.74, indicating acceptable classification ability. Notably, at its optimal cutoff, iron achieved perfect sensitivity, identifying all MS cases, although its moderate specificity suggests some misclassification of healthy controls. This pattern implies that elevated iron levels in the CC may represent an early or prominent pathological feature in MS, detectable even when other microstructural measures show limited discriminative value. In contrast, commonly used metrics such as T1, T2, and MTR showed AUC values close to chance, underscoring their limited utility in this cohort. The comparatively lower yet balanced discriminative performance of callosal volume further supports the relative strength of iron as a classifier. Overall, these results point to CC iron accumulation as a potentially sensitive indicator of MS-related tissue alterations, warranting further investigation into its mechanistic relevance and utility in diagnostic or monitoring frameworks.

Importantly, our results resonate with the growing body of evidence regarding paramagnetic rim lesions (PRLs), chronic active lesions in MS characterized by iron-laden, activated microglia/macrophages accumulating at lesion margins, visible on susceptibility-based MRI sequences. PRLs have emerged as robust in vivo biomarkers of chronic inflammation and ongoing tissue injury in MS [28]. These lesions have been linked to slower lesion expansion, greater risk of disability progression, and elevated neuroinflammatory activity [29]. While PRLs are typically described in focal white-matter lesions, their existence underscores a broader mechanism of iron dysregulation and microglial activation in MS. In this context, the diffuse iron accumulation observed in the CC, even in the absence of overt lesions, may represent a sub-clinical or early manifestation of similar pathogenic processes. Therefore, CC iron deposition might reflect a form of “diffuse smoldering” inflammation or iron-mediated vulnerability, paralleling mechanisms observed in PRLs, but affecting large fiber tracts rather than focal lesion margins. This expands the potential role of iron-sensitive MRI as a biomarker not only for focal lesion activity, but also for more widespread tissue involvement in MS. Further longitudinal studies will be necessary to assess whether CC iron levels predict future lesion development, atrophy, or clinical progression, much as PRLs have shown prognostic value in chronic MS [28–31].

The obvious decrease in the mean MTR values observed in the mid-posterior, central, and mid-anterior parts of the CC in RRMS patients compared to HCs can be due to different processes such as demyelination, axonal loss, and swelling, resulting in a reduction in the concentration of macromolecules in MS disease. In 1998, using a modest sample size (HC = 10, MS = 9) and a 1.5 T MRI scanner, Vavasour et al. reported reduced MTR values in the CC of MS patients compared to healthy controls, attributing these findings to pathological processes such as demyelination and axonal loss [32]. Conversely, a later study by Obberghen et al. in 2018, employing a relatively larger sample size (HC = 20, MS = 25) and imaging at 1.5 Tesla, found no significant differences in CC MTR values between MS patients and controls [33]. The discrepancies in these findings highlight the influence of methodological variations, including sample size and imaging protocols, on observed outcomes. Our study not only included a noticeably larger cohort (HC = 58, MS = 58), but also utilized advanced imaging techniques with a 3 Tesla MRI scanner. The higher magnetic field strength, combined with improved radiofrequency coil technology, significantly enhanced the signal-to-noise ratio and overall image quality, allowing for greater sensitivity in detecting subtle changes in MTR values. These methodological advancements, coupled with the larger sample size, provide a robust foundation for our findings, and accentuate the importance of employing state-of-the-art imaging protocols in studies of MS pathology. The observed negative associations between DD, EDSS scores, and MTR values in the CC emphasize the impact of MS progression on microstructural integrity. These findings support the notion that both prolonged disease duration and increasing disability are linked to deteriorating axonal integrity in the CC. Additionally, the negative correlation between white matter lesion volumes and MTR values further highlights the role of lesion burden in exacerbating microstructural damage. Together, these results suggest that MTR could serve as a sensitive marker for tracking disease progression and the extent of structural damage in the CC. An overarching challenge associated with MTR measurements is the absence of standardization across scanners and imaging protocols. This lack of uniformity complicates the comparison of findings across different centers unless additional quantification measures are concurrently employed [34].

The absence of significant differences in T1 relaxation times between RRMS and HCs in this study could be explained by the possibility that T1 prolongation (caused by demyelination and gliosis) was hampered by the increased iron deposition detected in most CC regions. Furthermore, a study by Sabine-Hofer et al. (2015) found spatial variations in T1 relaxation times within the human CC, hence, there is no common T1 relaxation time for the entire CC as it consists of regions with distinct and well-described fiber populations [35]. Most importantly, the observed positive correlations between T1 relaxation times in the CC and DD, EDSS scores, and white matter lesion volumes emphasize the potential of T1 relaxation as a marker for progressive pathological changes in MS. The robust correlations between T1 relaxation times and lesion volumes further support the role of remote white matter lesions in driving microstructural damage. These findings collectively suggest that T1 relaxation times can provide valuable insights into the spatial and temporal dynamics of MS pathology, particularly in regions most affected by disease duration.

In contrary to the T1 relaxometry, a significant increase in T2 relaxation times was detected only in the anterior CC of RRMS patients compared to HCs. This could be due to two competing processes occurring within the CC; demyelination and iron deposition. Demyelination in the CC fibers increases water content and diffusion, resulting in prolonged T2 relaxation times. In contrast, iron deposition enhances magnetic field inhomogeneity, accelerating signal decay and thereby shortening relaxation times. A study by Stevenson et al. (2000) reported a significant longer T2 relaxation time in the genu and splenium of the CC of MS patients than in HCs, although a relatively smaller sample size was used [36].

The positive correlations observed between T2 relaxation times and DD in various CC regions suggest that prolonged disease duration is associated with progressive microstructural changes in these areas. Similarly, the positive correlations between T2 relaxation times and EDSS scores underscore the link between increasing clinical disability and structural degradation. Additionally, the association between white matter lesion volumes and T2 relaxation times in CC regions highlights the sensitivity of T2 metrics to lesion burden and their potential as indicators of regional pathological involvement in MS. No significant associations were found between the T2 relaxation time of the CC and any of the clinical metrics in previous studies. In a single slice study, Whittall et al. (2002) could not identify significant correlation between patients’ clinical profiles and T2 relaxation times in the CC [37]. Similarly, Stevenson et al. (2000) reported no significant correlation was found between T2 relaxation time of the CC and participants’ EDSS [36].

The significant decrease in the CC volume of RRMS patients compared to HCs reflects underlying axonal loss and demyelination, particularly in interhemispheric fibers, which are hallmark pathological features of MS. The findings of this study reveal that CC atrophy is significantly associated with DD, disability, and white matter lesion load. Notably, weak to moderate negative correlations were observed between DD and CC volume across all CC regions, suggesting a widespread impact of disease progression on interhemispheric connectivity. In contrast, disability, as measured by EDSS scores, was only weakly associated with volume reductions in the mid-anterior and mid-posterior CC regions, indicating a more region-specific relationship between structural degeneration and functional impairment. Furthermore, the inverse relationship between white matter lesion load and CC volume across all CC regions reinforces the role of lesion burden in driving structural degeneration. These findings emphasize the utility of CC volumetric measures as region-specific biomarkers for tracking disease progression and clinical outcomes in MS. In support of these findings, Granberg et al. (2015), Platten et al. (2020), and Sotgiu et al. (2022) demonstrated a weak to moderate significant correlation between CC volume and EDSS, suggesting that CC atrophy is indeed associated with clinical disability [38–40]. However, the variability in the strength of the correlation across studies may reflect differences in study design, sample size, or imaging methods. Furthermore, Yaldizli et al. (2010) and Sugijono et al. (2020) explored the association between the CC index (CCI) and EDSS, rather than using CC volume [41, 42]. Both studies reported a negative association between the CCI and EDSS, indicating that lower CCI values were linked to higher levels of disability. These results further reinforce the importance of CC structural integrity as a potential marker for monitoring disability progression in MS.

The results highlight iron concentration as the most effective MRI-derived metric for distinguishing MS patients from healthy controls, with an AUC of 0.74 and perfect sensitivity, though its moderate specificity limits its standalone diagnostic utility. Volume showed more balanced performance, suggesting its potential for excluding healthy individuals. In contrast, T1, T2, and MTR metrics demonstrated poor discriminative ability, with AUCs near chance level and low specificities. These findings emphasize the importance of selecting robust imaging biomarkers and suggest iron and volume as promising candidates for clinical differentiation and disease monitoring in MS.

The quantitative results presented in this study indicate various interconnected pathological processes within the CC of RRMS patients. Nevertheless, these processes manifest with differing degrees of severity. Thus, it is imperative to expand this study by incorporating a larger sample size and encompassing different MS phenotypes.

### Conclusion

Quantitative MRI analysis revealed pronounced alterations in the CC of RRMS patients, with iron accumulation emerging as the most sensitive marker of disease-related changes. Among all MRI-derived metrics, iron demonstrated the highest discriminative performance, achieving perfect sensitivity, whereas volumetric measures provided complementary information with a more balanced diagnostic profile. These findings underscore the potential of CC iron as a biomarker for early detection, monitoring disease progression, and understanding iron-mediated mechanisms in MS, and support its integration into both clinical assessment and research applications.
